# Red Algal Sulfated Galactan Binds and Protects Neural Cells from HIV-1 gp120 and Tat

**DOI:** 10.3390/ph14080714

**Published:** 2021-07-23

**Authors:** Vitor H. Pomin, Fakhri Mahdi, Weihua Jin, Fuming Zhang, Robert J. Linhardt, Jason J. Paris

**Affiliations:** 1 Department of BioMolecular Sciences, School of Pharmacy, University of Mississippi, University, MS 38677-1848, USA; fmahdi@olemiss.edu; 2 Research Institute of Pharmaceutical Sciences, University of Mississippi, University, MS 38677, USA; 3 Center for Biotechnology and Interdisciplinary Studies, Department of Chemical and Biological Engineering, Rensselaer Polytechnic Institute, Troy, NY 12180, USA; jinweihua@zjut.edu.cn (W.J.); zhangf2@rpi.edu (F.Z.); linhar@rpi.edu (R.J.L.); 4 College of Biotechnology and Bioengineering, Zhejiang University of Technology, Hangzhou 310014, China; 5 Center for Biotechnology and Interdisciplinary Studies, Department of Chemistry and Chemical Biology, Rensselaer Polytechnic Institute, Troy, NY 12180, USA

**Keywords:** glycoprotein 120, HIV, neuroprotection, surface plasmon resonance, sulfated glycan, trans-activating transcriptor

## Abstract

The potential neuroprotective capacity of four different sulfated glycans: *Botryocladia occidentalis*-derived sulfated galactan (BoSG) (MW > 100 kDa), *Lytechinus variegatus*-derived sulfated fucan (LvSF) (MW~90 kDa), high-molecular weight dextran sulfate (DxS) (MW 100 kDa), and unfractionated heparin (UFH) (MW~15 kDa), was assessed in response to the HIV-1 proteins, R5-tropic glycoprotein 120 (gp120) and/or trans-activator of transcription (Tat), using primary murine neurons co-cultured with mixed glia. Compared to control-treated cells in which HIV-1 proteins alone or combined were neurotoxic, BoSG was, among the four tested sulfated glycans, the only one capable of showing significant concentration-dependent neuroprotection against Tat and/or gp120, alone or combined. Surface plasmon resonance-based data indicate that BoSG can bind both HIV-1 proteins at nM concentrations with preference for Tat (7.5 × 10^−8^ M) over gp120 (3.2 × 10^−7^ M) as compared to UFH, which bound gp120 (8.7 × 10^−7^ M) over Tat (5.7 × 10^−6^ M). Overall, these data support the notion that sulfated glycan extracted from the red alga *B. occidentalis*, BoSG, can exert neuroprotection against HIV-1 Tat and gp120, potentially via direct molecular interactions.

## 1. Introduction

Human immunodeficiency virus type 1 (HIV-1) infection is associated with neurological symptomatology, including cognitive deficits, affective disorders, and motor impairment [1]. Neurological symptoms, collectively termed ‘neuroHIV’, persist despite treatment with combined antiretroviral therapeutics (cART), likely due to poor accumulation of cART within the central nervous system where viral neurotoxins promote inflammation and neuronal damage [2,3]. The most well-characterized neurotoxic HIV-1 proteins include the HIV-1 envelope protein, glycoprotein 120 (gp120), and the HIV-1 regulatory protein, trans-activator of transcription (Tat). Through shared and separate neuroinflammatory and excitotoxic mechanisms, gp120 and Tat promote synaptodendritic damage and neuronal death [4,5,6]. Expression of either protein is enough to recapitulate many aspects of neuroHIV in rodent models [7,8]. Thus, the identification of centrally active and safe therapeutics that can ameliorate gp120/Tat-mediated neurotoxicity is important for the improvement of current cART strategies.

Heparan sulfate proteoglycans (HSPGs), a key component of the extracellular matrix, are known to bind and regulate four different HIV-1 proteins: gp120 [9,10,11,12], Tat [12,13,14,15,16,17,18], HIV matrix protein p17 (MA) [19,20,21], and glycoprotein 41 (gp41) [22]. However, HSPG interactions for two of these proteins, gp120 and Tat, are more well-characterized than the others. As such, sulfated glycans may be developed as novel anti-HIV therapeutics, especially analogs of similar or different structures that could compete with HSPG [12,23]. Given the already identified HS (heparan sulfate) interactions with HIV-1 gp120 and Tat and the importance of these proteins for viremia, latency, and subsequent pathologies, prior research has suggested the development of various potential anti-HIV sulfated glycans, including glycosaminoglycans (GAGs) and those of marine origin [12,24].

Herein, we evaluate the potential neuroprotective properties of four structurally distinct sulfated glycans against the HIV-1 proteins gp120 and Tat. We examined unfractionated heparin (UFH) [25], 100 kDa-dextran sulfate (DxS(100)) [26], the tetrasaccharide repeating sulfated fucan isolated from the sea urchin *Lytechinus variegatus* (LvSF) [27], and the disaccharide repeating sulfated galactan isolated from the red alga *Botryocladia occidentalis* (BoSG) [28]. We conducted an in vitro live/dead cell-based assay using co-cultured primary striatal medium spiny neurons and mixed glia obtained from mice, as well as a binding assessment using surface plasmon resonance (SPR) to begin to assess the neuroprotective capacity of these compounds and their potential interactions with HIV-1 proteins. We anticipated the sulfated glycans to ameliorate gp120 and Tat-mediated neurotoxicity and to bind these proteins directly. Furthermore, we suggest that the present study yields new insights on the sulfated glycan structural components that are important for HIV interactions.

## 2. Results

### 2.1. Structure and Selection of Sulfated Glycans

**Figure 1:**
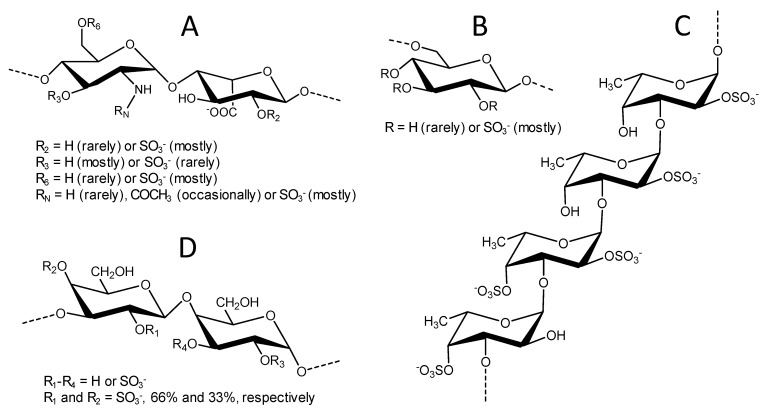
Structural representation of unfractionated heparin, UFH (**A**); dextran sulfate, DxS (**B**); *Lytechinus variegatus* sulfated fucan, LvSF (**C**); and *Botryocladia occidentalis* sulfated galactan, BoSG (**D**).

The chemical structures of the four sulfated glycans utilized in the live/dead cell-based assay and SPR studies are displayed in Figure 1. Although structurally complex, UFH (15 kDa) is largely composed of the disaccharide repeating units of [-4)-*N*,6-disulfated-glucosamine-(α1-4)-2-sulfated-iduronic acid-(α1-] (Figure 1A). Other minor heterogeneities can be also found in its chain, such as the rare *O*-sulfation at C3 position, the rare *N*-unsubstitution of the amino group (*N* free, *N*H), and the occasional occurrence of *N*-acetylation (*N*HCOCH_3_) in the glucosamine unit, as well as the lack of 2-*O*-sulfation in the iduronic acid unit (Figure 1A). DxS(100) (100 kDa) is less heterogeneous and composed of [-6)-glucose-(α1-] units heavily sulfated at positions C2, C3, and C4 (Figure 1B). LvSF (~90 kDa) is composed of the tetrasaccharide-repeating unit of [-3)-4-sulfated-fucose-(α1-3)-2,4-disulfated-fucose-(α1-3)-2-sulfated-fucose-(α1-3)-2-sulfated-fucose] (Figure 1C). Finally, BoSG (>100 kDa) is composed of disaccharide repeating units of [-3)-2,4-disulfated-galactose-(α1-4)-2,3-disulfated-galactose-(β1-], whose sulfation patterns may vary in proportion but never in position (Figure 1D). From this set of structurally distinct sugars, we expected to be able to identify the structure(s) that best confer binding and protection in the presence of the HIV-1 proteins. Chemical features of these sulfated glycans, such as oligosaccharide length of the building block (monosaccharide, disaccharide, or tetrasaccharide), monosaccharide composition (glucosamine and iduronic acid, glucose, galactose, or fucose), different glycosidic linkages (at C3, C4, and C6 positions), sulfation patterns (*N*- or *O*-positions of C2, C3, C4), and molecular weight (MW) may confer distinct functional effects. This justifies an advanced structure–activity relationship study using more homogenous or defined sulfated glycans, such as those from marine origins.

### 2.2. Neuroprotective Capacity of Marine and Control Sugars against HIV-1 Proteins

**Figure 2:**
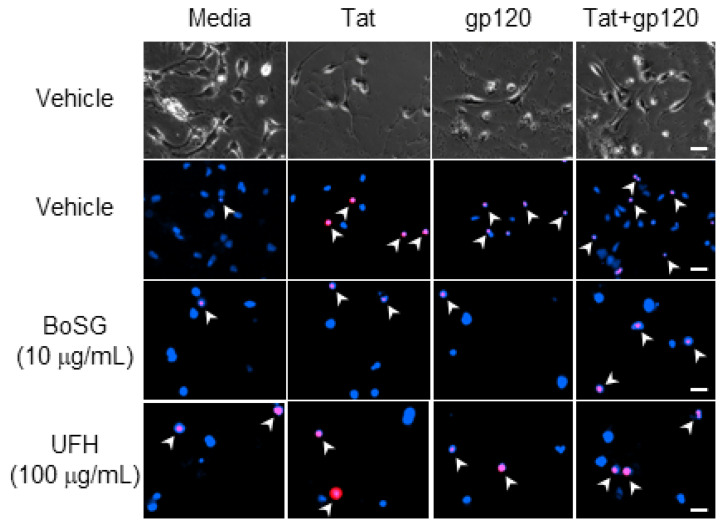
Representative photomicrographs (40×) of C57BL/6N, striatal, medium spiny neurons co-exposed to *Botryocladia occidentalis* sulfated galactan (BoSG) or unfractionated heparin (UFH) and the HIV proteins, Tat (50 ng/mL) and/or gp120 (500 pM). Total cell number was assessed via Hoechst 33342 (blue) and cell death was confirmed via propidium iodide (red). Arrows indicate examples of propidium-iodide-positive cells. Scale bar = 20 µM.

**Figure 3:**
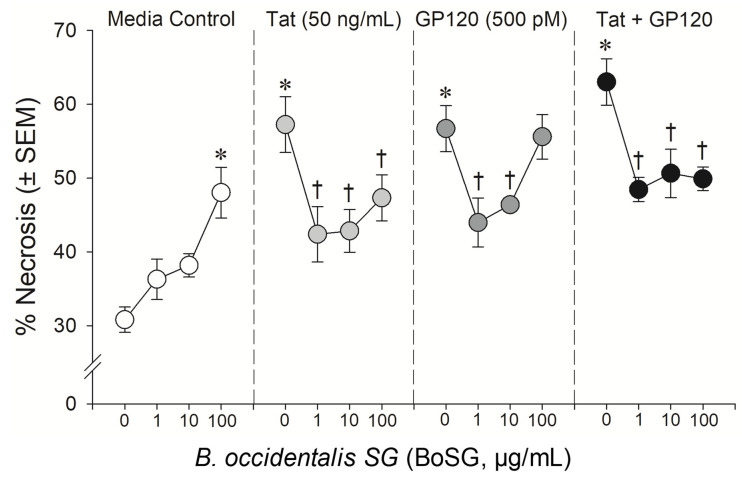
Neuronal cell death among C57BL/6N, striatal, medium spiny neurons co-exposed to *Botryocladia occidentalis* sulfated galactan (BoSG; 1, 10, and 100 μg/mL), HIV-1 Tat (50 ng/mL), and/or HIV-1 gp120 (500 pM). * indicates significant increase in necrosis compared to untreated media control. † indicates significant protection from respective HIV-1 protein exposure. All points indicate the mean of 3–5 independent experiments (two-way ANOVA, *p* ≤ 0.05).

**Figure 4:**
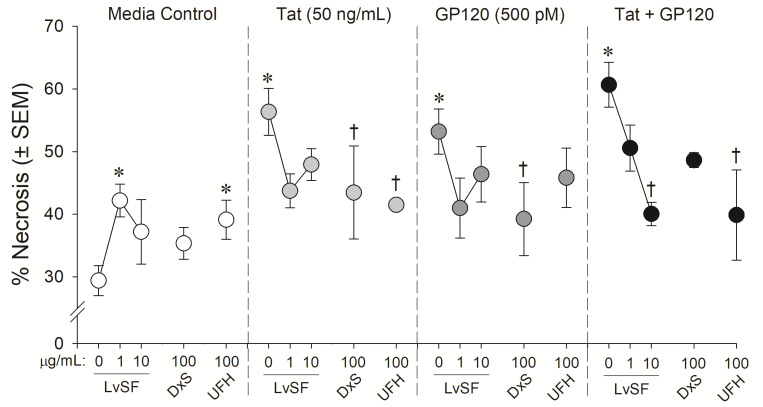
Neuronal cell death among C57BL/6N, striatal, medium spiny neurons co-exposed to *Lytechinus variegatus*-derived sulfated fucan (LvSF; 1 and 10 μg/mL), high-molecular weight dextran sulfate (DxS; 100 µg/mL), unfractionated heparin (UFH; 100 µg/mL), HIV-1 Tat (50 ng/mL), and/or HIV-1 gp120 (500 pM). * indicates significant increase in necrosis compared to untreated media control. † indicates significant protection from respective HIV-1 protein exposure. All points indicate the mean of 2–5 independent experiments (two-way ANOVA, *p* ≤ 0.05).

The anti-Tat and anti-gp120 neuroprotective capacity of the marine sugars, BoSG and LvSF, were assessed in a concentration-response manner (1, 10, and 100 μg/mL BoSG and 1 and 10 μg/mL LvSF), while control sugars, DxS(100) and UFH, were assayed only at the highest concentration of 100 μg/mL (Figure 2, Figure 3 and Figure 4). Exposing neural co-cultures to HIV-1 Tat and/or gp120 promoted neurotoxicity and the marine sugar BoSG exerted significant protection (*F*(9,44) = 4.80, *p* = 0.0002) (Figure 2 and Figure 3). Compared to control-treated cells, Tat (*p* < 0.0001), gp120 (*p* < 0.0001), or combined Tat and gp120 (*p* < 0.0001) significantly increased neuronal death (Figure 3). While BoSG demonstrated increased neurotoxicity at the highest concentration on its own (100 µg/mL; *p* = 0.0003), it also significantly protected against the HIV-1-mediated neurotoxicity caused by Tat at any concentration assessed (*p*_1µg/mL_ = 0.01, *p*_10µg/mL_ = 0.02, *p*_100µg/mL_ = 0.05), caused by gp120 at 1 (*p* = 0.01) or 10 µg/mL (*p* = 0.03), or caused by combined Tat/gp120 at any concentration assessed (*p*_1µg/mL_ = 0.003, *p*_10µg/mL_ = 0.008, *p*_100µg/mL_ = 0.003; Figure 3).

Similarly, the marine sugar, LvSF, and control sugars exerted some significant neuroprotection from HIV-1 proteins exposure (*F*(12,40) = 2.44, *p* = 0.02) (Figure 2 and Figure 4). Compared to control-treated wells, HIV-1 proteins again significantly increased neurotoxicity (*p*_Tat_ < 0.0001; *p*_gp120_ = 0.0001; *p*_Tat/gp120_ < 0.0001; Figure 4). LvSF exerted some dose-dependent toxicity on its own at 1 µg/mL (*p* = 0.02) but not 10 µg/mL, as did UFH (*p* = 0.03). LvSF exerted significant neuroprotection only against combined Tat and gp120 at the highest concentration (*p* = 0.01; Figure 4). DxS exerted significant neuroprotection against HIV-1 Tat (*p* = 0.05) or gp120 (*p* = 0.04) alone, but not the combination of both (Figure 4). UFH only significantly protected against Tat (*p* = 0.03) or combined Tat and gp120 (*p* = 0.005; Figure 4).

### 2.3. Binding Capacity of BoSG and UFH of HIV-1 Proteins

**Figure 5:**
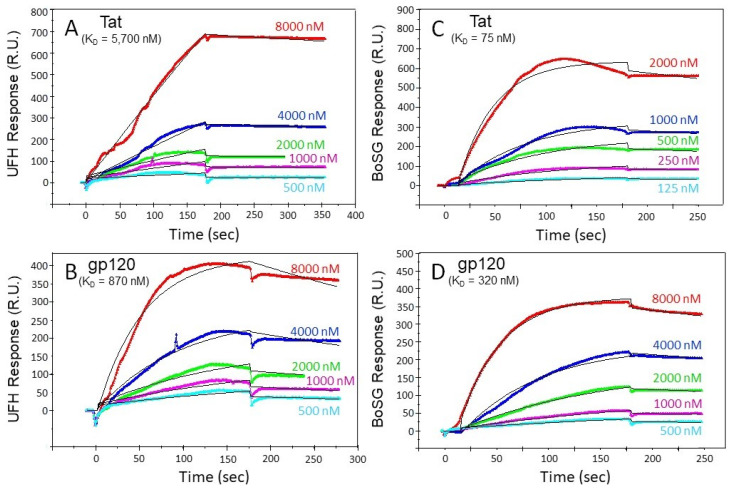
SPR sensorgrams of unfractionated heparin UFH; (**A**,**B**) and *Botryocladia occidentalis* sulfated galactan BoSG; (**C**,**D**) in interactions with HIV-1 Tat (**A**,**C**) and gp120 (**B**,**D**). Concentrations of HIV-1 proteins are shown color-coded in the panels. The black curves are the fitting curves using models from BIAevaluate 4.0.1.

In follow-up to the functional effects for BoSG’s neuroprotection against HIV-1 Tat and/or gp120, SPR measurements were performed to assess the individual binding affinities of BoSG for either protein. Data were compared to UFH as a well-known standard (Figure 5). After biotinylation (reaction described in Materials and methods), both BoSG and UFH were immobilized on a high-affinity streptavidin (SA) sensor chip. Then, the HIV-1 proteins were injected to the flow cell of a SA chip at a flow rate of 10 μL/min at different final nanomolar concentrations (see color-coded curves). Binding measurements were conducted at 25 °C. Successful immobilization of the sugars could be confirmed by a ~200 resonance unit (RU) increase in the sensor chip. The resultant binding kinetics were demonstrated by the curves of response difference as a function of time and protein concentrations in the sensorgrams. From these sensorgrams, kinetic values such as association rate constant (ka), dissociation rate constant (kd), and dissociation constants (*K*_D_) for the four intermolecular complexes were generated (Table 1).

The SPR-derived results have indicated that BoSG can bind to both HIV proteins at similar molar ranges of heparin, between tens of nanomolar to a few micromolar concentration. BoSG bound tighter to Tat (7.5 × 10^−8^ M) compared to UFH-Tat (5.7 × 10^−6^ M), UFH-gp120 (8.7 × 10^−7^ M), and BoSG-gp120 (3.2 × 10^−7^ M) (Table 1). These data indicate that BoSG presents higher specificity of interaction with Tat than gp120, and this supports the observation from the cell-based assay (Figure 2 and Figure 3). The result from the SPR study also supports the finding that BoSG presents stronger affinity for both HIV-1 proteins than UFH (two orders of magnitude in the case of Tat and almost three-fold more in the case of gp120), justifying the necessity of lower concentrations of BoSG to inhibit the neurotoxic effects of the HIV proteins in the cell-based assay (Table 1). As confirmed by SPR, results have significantly indicated stronger, although not very pronounced, affinity of BoSG for the HIV-1 proteins as compared to UFH.

## 3. Discussion

The present data upheld the hypothesis that sulfated glycans are a class of natural products that can functionally ameliorate HIV-1 Tat and/or gp120-mediated neurotoxicity. In the current study, a set of four different sulfated glycans were used: UFH as a standard control as widely exploited in many pharmacological works investigating sulfated glycans and/or GAGs [25]; DxS(100) as the second control since this particular sulfated glycan has been widely investigated in the past regarding its anti-HIV activity [12], and the two marine unique compounds: the sea-urchin-derived sulfated fucan from *L. variegatus*, LvSF [27] and the sulfated galactan derived from the red alga *B. occidentalis*, BoSG [28]. These two marine sulfated glycans show different chemical and biological features. The LvSF is regular and defined in terms of structure and lacks biological effect as an anticoagulant and antithrombotic agent, while BoSG is more sulfated and heterogeneous; and exhibits great effects when investigated as an anticoagulant and antithrombotic agent [29]. Hence, structure–function relationship studies in neuroHIV can be achieved using this set of four structurally and functionally different sulfated glycans. Compared to the other tested sugars, the major structural difference in BoSG is that it is composed solely of galactose units.

Overall, data reveal BoSG to exert greater neuroprotection compared to the controls, DxS(100) and UFH. Importantly for therapeutic consideration, this red algal sulfated galactan demonstrates no increased bleeding effects as opposed to the well-documented hemorrhagic side-effects of DxS and UFH after intravenous administration [30]. DxS and UFH are also known to present thrombocytopenic effects in treatments [31,32], including those in HIV therapy [32]. As opposed to LvSF, which showed significant neuroprotective action only against the proteins combined at the highest concentration, BoSG was capable of significantly inhibiting in a concentration-dependent manner both HIV proteins in all tested conditions, proteins alone and/or combined. The decision to conduct further SPR analyses compared to the control UFH was made, therefore, in favor of only BoSG. Data from SPR confirm preferential binding of BoSG with Tat over gp120. Together, the data from this investigation provide new insights in the consideration of the marine sulfated glycans, particularly the red algal sulfated galactan, BoSG, for their capacities to interact with and ameliorate HIV-1 protein neurotoxicity.

Previously, the role of HIV-1 gp120 in the binding of host cell HSPGs was identified as a key process for infection [9,11]. Several sites of molecular interaction may mediate these effects. HSPG binding is partly modulated by a conformational change in gp120 that is induced through its interaction with CD4 [11]. The V3 loop (CD4-induced epitope) of gp120 is essential for binding to HS and for the initial process of T-cell infection [9]. The V3 loop contains a binding domain for CD4 that, when activated, induces a conformational change that is necessary for gp120–HSPG interactions to occur [9]. As such, the HSPG interaction is thought to follow the initial step of gp120 binding to CD4 during viral-cell attachment [9].

Through residue mapping experiments using mutagenesis and SPR spectroscopy, the gp120 amino acids Arg-419, Lys-421, and Lys-432 have been identified to be highly important for the binding of HS in the presence of CD4 [11]. These positively charged amino acids comprise the typical binding site of GAG-binding proteins [33]. In addition to this GAG binding site, other binding sites were also mapped and found to comprise the V2 loop, a portion of the C-terminal, and the CD4-induced bridging sheet, together with the V3 loop [11]. Interestingly, three of these gp120-GAG-binding sites undergo conformational change upon interaction with CD4 and are directly involved in co-receptor recognition [11]. These data suggest that gp120-HSPG-binding sites interact with HIV-1 co-receptors, making them an intriguing target for novel HIV-1 entry inhibitors.

Molecular interactions between HIV-1 Tat and HSPGs are also likely to occur. Following infection, transcribed HIV-1 Tat acts on the HIV-1 LTR, greatly increasing the efficiency of viral transcription. However, as a soluble protein that can be secreted from infected cells, Tat also exerts effects on the extracellular environment and nearby uninfected cells that further catalyze HIV infectivity through multiple mechanisms [34]. Tat may bind to a component of the extracellular milieu, the HSPG, to increase its effective concentration at the cell-surface and to gain access to nearby uninfected cells [14]. Tat–HSPG interactions may occur via binding of the basic region of Tat, serving as the HS (or GAG) binding domain [14]. This prior work suggested that HS was responsible for the attachment, sequestration, and retention of Tat at the cell surface, but the molecular player(s) responsible for cellular uptake were not known until the discoveries made 4 years later. These findings revealed HS acts as a cell membrane surface receptor for Tat and its consequent cellular internalization [16]. Furthermore, the binding and uptake of Tat can be prevented by using competitive soluble heparin or GAG lyases that can cleave the HS chain [16]. These data support a role for HS in HIV-1 viremia.

Given demonstrations of HS binding to and internalizing Tat during the HIV infection, many other works have appeared in the literature discussing the aspects of the HS–Tat interactions and their biochemical consequences in HIV/AIDS [13,15,17,18]. Among these, two of great relevance include demonstrations that (1) the binding and clustering of Tat molecules by HSPGs occurs at low-micromolar concentrations [18] and (2) a quaternary complex containing Tat homodimers with two HSPGs (HS/Tat–Tat/HS) are physiologically assembled in order to promote adhesion, migration, and extravasation of lymphocytes across the endothelium during HIV infection [17]. The current results provide evidence that the marine sugars, particularly BoSG, can efficiently compete with HSPGs required for HIV neuroinfection. 

Future in vivo studies to assess efficacy and safety of BoSG must be also conducted to advance the status of this marine sulfated glycan as a potential drug candidate in phase I clinical trials. Other open questions may be related to bioavailability and to how this marine molecule can efficiently reach the central nervous system (CNS) for the desired effects. Data from the literature have demonstrated that BoSG to exert venous antithrombotic actions when intravenously administered [35]. This observation supports the notion that BoSG can be bioactive in the body. Although the blood–brain barrier (BBB) permeability for native BoSG and low MW derivatives is virtually unknown, data exist in the literature demonstrating low MW heparin derivatives to efficiently pass through the BBB [36]. The production and exploitation of low MW BoSG derivatives may prove to be a promising strategy to increase the efficacy and bioavailability of BoSG in the CNS. Melo et al. have already shown the paths for production and biomedical use of such BoSG oligosaccharides [37]. Future investigations aimed to assess the influence of native BoSG and its derived oligosaccharides on HIV-1 viremia are also required, as are investigations on the effects at other cell types.

## 4. Materials and Methods

### 4.1. Chemical, Reagents and Instruments

Recombinant HIV proteins gp120_ADA_ (product# 1081) and Tat_1-86_ (product# 1002-2) were purchased from ImmunoDiagnostics, Inc. (Woburn, MA, USA). Heparin sodium salt from intestinal mucosa (CAS Number: 9041-08-1), dextran sulfate sodium salt from *Leuconostoc spp.* (CAS Number: 9004-54-0), and papain (CAS Number: 9001-73-4) were purchased from Sigma Aldrich (Saint Louis, MO, USA). Sensor SA chips were purchased from GE healthcare (Uppsala, Sweden). DEAE was purchased from Santa Cruz Biotech (Santa Cruz, CA, USA). SPR measurements were performed on a BIAcore 3000 operated using BIAcore 3000 control and BIAevaluation software (version 4.0.1). The marine organisms red alga *B. occidentalis*, and the sea urchin *L. variegatus* were collected and identified as previously described [27,28].

### 4.2. Sulfated Glycans

Purification of BoSG and LvSF were conducted as previously described [27,28]. Briefly, 20 mg of crude polysaccharides obtained after papain digestion were extracted from the respective tissues (red algal body wall and sea urchin egg jelly) and applied to a DEAE-cellulose column (2.5 cm diameter × 20 cm width) that was equilibrated with 50 mM sodium acetate buffer (pH 5.0) and washed with 300 mL of the same buffer containing 0.2 M NaCl and 10 mM EDTA. Thereafter, the column was eluted by a linear gradient prepared by mixing 2000 mL of 50 mM sodium acetate buffer (pH 5.0) containing 0.2 M NaCl and 10 mM EDTA with 200 mL of 1.2 M NaCl in the same buffer. The flow rate of the column was 12 mL/h. Fractions of 1.5 mL were collected and checked by metachromatic properties [38]. The chromatograms of the two materials resulted in three major fractions. The third fraction of each chromatography corresponded to BoSG and LvSF. Prior to submission of samples, either for cell-based in vitro assay or SPR studies, the purity level and the structural integrity of BoSG and LvSF were checked by 1D ^1^H (400 MHz) NMR spectra acquired with 32 scans at the 400 MHz Bruker Avance III HD equipped with 5 mm BBFO RT probe. The resultant NMR spectra indicated purity of >98%, no residual contamination from solvents, and an NMR signal pattern as expected with the previous publications [27,28]. For NMR analysis, ~5 mg of sugar samples were individually dissolved in 550 μL of deuterium oxide (D_2_O) “100%” (D 99.96%), purchased from Cambridge Isotope Laboratories, Inc. (Andover, MA, USA), and transferred to a 3 mm NMR tube for data acquisition. The NMR spectra, as well as the polyacrylamide gel electrophoresis for assessment of MW distribution of the two marine sample preparations, BoSG and LvSF, can be found in [39], in which the same sample preparations were studied.

### 4.3. Live/Dead Neural Cell-Based Assay

#### 4.3.1. Neural Cell Co-Culture

Neuron-glial co-cultures were prepared as previously described [40,41]. In brief, primary mixed-glia cultures were derived from the striatum of postnatal day 0–1 C57BL/6N mice. Dissected striata were minced, incubated (37 °C, 5% CO_2_) with trypsin (2.5 mg/mL; #T4799, Sigma-Aldrich, Saint Louis, MO, USA) and DNase (15 µg/mL; #D5025, Sigma-Aldrich) in Dulbecco’s Modified Eagle’s Medium (DMEM) for 30 min, triturated, and filtered through 100-μm then 40-μm diameter pore cell strainers (Greiner Bio-One, Kremsmünster, Austria). Mixed glia were plated at a density of 500,000 cells/well onto poly-L-lysine-coated (#P2636, Sigma Aldrich) 12-well culture plates and maintained for 4 days in DMEM supplemented with 10% fetal bovine serum. Primary striatal neurons cultured from embryonic day 14–15 mouse pups were dissociated similar to glia, with the exception that 70-μm diameter pore cell strainers (Greiner Bio-One, Kremsmünster, Austria) were used. Following isolation, neurons were seeded at a density of 30,000 cells/well onto previously established 4-day-old mixed-glia cultures. Neuron-glia co-cultures were maintained in neurobasal medium (#21103049; Life Technologies) supplemented with B27 (#12587010, Life Technologies, Carlsbad, CA, USA), L-glutamine (9.5 mM; Life Technologies), glutamate (25 mM; Sigma-Aldrich), and an antibiotic mixture. Co-cultures were maintained in a humidified incubator (37 °C, 5% CO_2_) for 7–8 days prior to assay.

#### 4.3.2. Live/Dead Assay

Neuron viability was assessed using a LIVE/DEAD^®^ Viability/Cytotoxicity Kit (#L-7013, Molecular Probes, Eugene, OR, USA) per manufacturer instructions and as described [42,43]. Briefly, experimental treatments (sulfated glycans and/or HIV-1 proteins) were applied to primary co-cultures and live/dead assay was performed 20 h post treatment. Prior work utilizing time-lapse microscopy (0–60 h) identified the 20 h time-point as the earliest time when Tat-treated cells significantly diverged on the measure of viability [41]. A working solution of propidium iodide (ex/em: 535/617 nm) and Hoechst 33342 (ex/em: 360⁄460 nm) was prepared by diluting the stock solutions in Hank’s Balanced Salt Solution containing Ca^2+^ and Mg^2+^ (HBSS; 1:50 and 1:10,000 dilution, respectively). Cell media was replaced with the propidium iodide/Hoechst 33342 working solution and incubated (37 °C; 5% CO_2_) for 15 min in the dark. Cells were washed three times with HBSS and imaged on a Nikon Ti2-E inverted microscope (Nikon, Melville, NY, USA) with a motorized stage. Cells were quantified from images captured at 40 × magnification (*S* Plan Fluor, 0.6 NA). Viability was assessed by calculating the proportion of necrotic cells (# propidium iodide + cells/# Total Cells) × 100).

#### 4.3.3. Statistical Analyses

Cell death was separately analyzed for BoSG and additional sulfate glycans by two-way ANOVA with compound and concentration as the between subjects’ factors. Fisher’s protected least significant difference post-hoc tests determined group differences following main effects. Interactions were delineated via simple main effects and main effect contrasts with alpha controlled for multiple comparisons. Analyses were considered significant when *p* ≤ 0.05.

### 4.4. SPR

#### 4.4.1. Preparation of BoSG and UFH Biochip

UFH or BoSG (2 mg each) and amine-PEG3-Biotin (2 mg, Pierce, Rockford, IL, USA) were dissolved in 200 µL H_2_O, followed by addition of 10 mg NaCNBH_3_ to the solution. The reaction mixture was heated at 70 °C for 24 h and followed by further addition of 10 mg NaCNBH_3_.The reaction mixture was heated at 70 °C for an additional 24 h. After cooling to room temperature, the mixture was desalted with the spin column (3000 MWCO). Biotinylated BoSG and UFH were collected, freeze-dried, and used for SA chip preparation. The biotinylated BoSG and UFH were immobilized to streptavidin (SA) chip based on the manufacturer’s protocol. In brief, 20 µL solution of biotinylated BoSG (0.1 mg/mL) or 40 µL solution of biotinylated UFH (2 mg/mL) in HBS-EP running buffer was injected over flow cell 2 (FC2) of the SA chip at a flow rate of 10 µL/min. The successful immobilization of these sulfated glycans was confirmed by the observation of a ~200 to 400 resonance unit increase in the sensor chip. The control flow cell (FC1) was prepared by 1 min injection with saturated biotin.

#### 4.4.2. Measurement of Interaction between Sulfated Glycans and Proteins Using BIAcore

The HIV-1 proteins (Tat or gp120) were diluted in HBS-EP buffer (0.01 M HEPES, 0.15 M NaCl, 3 mM EDTA, 0.005% surfactant P20, pH 7.4). Different dilutions of protein samples were injected at a flow rate of 40 µL/min. At the end of the sample injection, the same buffer was flowed over the sensor surface to facilitate dissociation. After a 3 min dissociation time, the sensor surface was regenerated by injection of 40 µL of 2 M NaCl. The response difference (RU) was monitored as a function of time (sensorgram) at 25 °C. The resulting sensorgrams were used for interaction kinetics and binding affinity determination: association rate constant: ka; dissociation rate constant: kd; and binding equilibrium dissociation constant: K_D_, K_D_ = kd/ka, by global fitting using 1:1 Langmuir binding model from Biaevaluation software 4.0.1 (GE healthcare, Uppsala, Sweden).

## 5. Conclusions

The HIV-1 proteins gp120 and Tat are required for viral infection and replication of the virus. In addition, they may promote neurotoxicity despite cART treatment. Heparan sulfate or heparin are known to be neuroprotective against these two HIV proteins. Herein, we identify the neuroprotective effects of four different sulfated glycans and reveal a sulfated galactan derived from the red alga *B. occidentalis* as a compound with potential for the treatment of HIV-1 Tat/gp120-mediated neurotoxicity. Compared to the other tested sugars, the major structural difference in BoSG is that it is composed solely of galactose units, while UFH, DxS(100), and LvSF are composed of hexosamine and hexuronic acid, glucose, or fucose respectively. Sulfated galactans of different structures, such as the homogenous sulfated galactan from the sea urchin *Glyptocidaris crenularis* [44] or the 3-linked 4-sulfated galactan derived from *Codium isthmocladum* [45], remain to be investigated in comparison to sugars of different monosaccharide compositions. Further study is needed to confirm the neuroprotective effect of the galactose-composed polysaccharides against HIV-1 proteins. Moreover, the anti-viremia capacity of BoSG and its potential interactions with cART remain questions for future investigation.

## Figures and Tables

**Table 1:** Kinetic values from the molecular interactions between UFH and BoSG with Tat and gp120.

Interaction	*k*_a_ (1/MS)	*k*_d_ (1/S)	*K*_D_ (M)
UFH + Tat	48 ± 3.4	2.7 × 10^−4^ ± 2.0 × 10^−5^	5.7 × 10^−6^
UFH + gp120	2.2 × 10^3^ ± 64	1.9 × 10^−3^ ± 9.2 × 10^−5^	8.7 × 10^−7^
BoSG + Tat	1.4 × 10^4^ ± 237	1.0 × 10^−3^ ± 1.1 × 10^−4^	7.5 × 10^−8^
BoSG + gp120	3.1 × 10^3^ ± 30	9.8 × 10^−4^ ± 5.9 × 10^−5^	3.2 × 10^−7^

Standard deviation values, after ± symbol, were obtained from global fitting of five different injections using a 1:1 Langmuir model.

## Data Availability

The data presented in this study are available on request from the corresponding authors in adherence with guidance from the U.S. Department of Health and Human Services, the National Institutes of Health.
